# Electro-spinning of highly-aligned polyacrylonitrile nano-fibres with continuous spooling

**DOI:** 10.1038/s41598-021-99890-w

**Published:** 2021-11-05

**Authors:** Siheng Shao, Tao Ma, Gerard F. Fernando

**Affiliations:** Sensors and Composites Group, School of Metallurgy and Materials, University of Birmingham, Edgbaston, Birmingham, B15 2TT UK

**Keywords:** Nanoscale materials, Structural materials, Techniques and instrumentation

## Abstract

This paper reports on a new configuration for producing highly-aligned electro-spun fibres that can be produced on a static substrate or one where it is hauled off and spooled continuously to enable the production of continuous lengths. The fixture consists of a Vee-shaped polytetrafluorethylene shield at 60° with a 1 cm wide integral rectangular base that is mounted on a copper disk with a 10 cm diameter. Specified concentrations of polyacrylonitrile in dimethyl sulfoxide were electro-spun on to a strip of cellulose paper. In the static setup, approximately 91% of the fibres were deposited to within 3°. When the spooling rig was used, a tape of the cellulose paper was hauled off at 0.07 mm/min, 78% of the fibres were aligned to within 3°. Simulations of the conventional and Vee-shield electro-spinning setups were undertaken and they provided corroboration for the experimental observations with regard to the mechanism responsible for fibre alignment. The feasibility of using this technique to produce 0°/− 45°/+ 45° stacked layers of aligned fibre preform is demonstrated.

## Introduction

**Figure 1:**
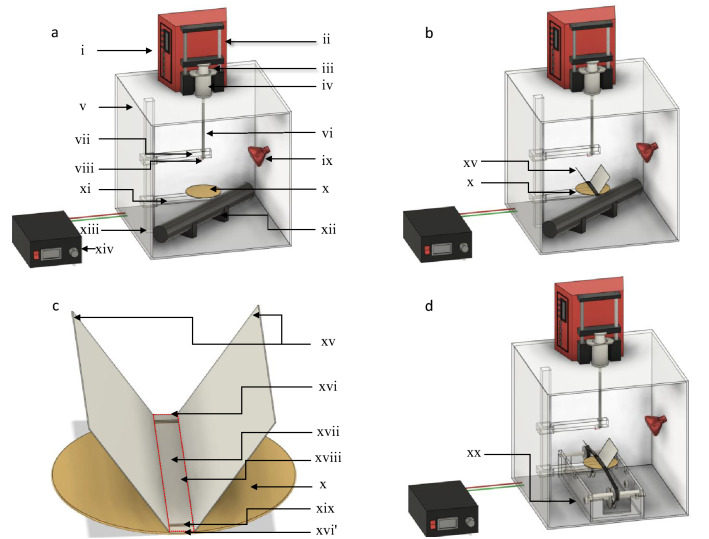
(**a**) Schematic illustration of a conventional electro-spinning setup; (**b**) the new Vee-shield experimental setup; (**c**) a magnified view of the Vee-shield; and (**d**) the motorised spooling rig for continuous production of aligned fibres. The coded items are as follows: (i) controller for the liquid dispensing system; (ii) screw-driven drive mechanism on the liquid dispenser; (iii) plunger of the syringe; (iv) barrel of the syringe; (v) Perspex chamber (with doors); (vi) PTFE tube that connects the tip of the syringe to the needle (via Luer Lock connectors); (vii) Perspex support arm for the needle; (viii) metal needle; (ix) infrared heat source; (x) circular grounded metal collector; (xi) Perspex support arm for the grounded collector; (xii) storage heater; (xiii) vertical Perspex support arm; (xiv) high-voltage DC power supply; (xv) PTFE Vee-shield; (xvi and xvi’) back and front edges of the ground electrode respectively; (xvii) strip of cellulose paper; (xviii) perimeter where the majority of the aligned fibres are deposited; and (xix) slot in the PTFE where the cellulose substrate is fed through when spooling (a similar slot is present on the opposite end); (xx) motorised spooling rig for the continuous production of aligned electro-spun fibres.

Electro-spinning is an established manufacturing technique for the production of fibres with diameters typically in the nanometre and micrometre range. A number of materials have been electro-spun including polymers^1^, polymer blends^2^, polymer systems involving ceramic and metallic precursors^3,4^ and precursor-based compounds for subsequent conversion via oxidation or reduction^5^. The technique is comparatively straightforward when compared to other methods for producing reinforcing fibres with a circular cross-section and without any fused fibres. A schematic illustration of a conventional electro-spinning set up is shown in Fig. 1a. It consists of a precision liquid dispensing system that drives the plunger of a syringe with a metal needle. The needle is connected to a high-voltage power supply and a conductor is used as the grounded plate where the fibres are deposited during electro-spinning. The mode of operation of conventional electro-spinning is as follows.

With reference to Fig. 1a, once the polymer solution has been introduced into the syringe with a needle, and secured in position, the liquid dispenser or pump is set to deliver a constant volume to maintain a pendant drop at the tip of the needle during electro-spinning. The initial shape of the pendant drop can be described as a sphere. As the applied voltage is increased slowly, the magnitude of the Coulombic repulsion forces within the polymer solution increases. When the applied voltage is increased further, the shape of the pendant drop starts to deform, and it takes a conical profile that is generally referred to as the Taylor cone^6^. There comes a point, as the applied voltage is increased, the repulsive forces within the polymer solution overcome the surface tension of the liquid and a solvent-loaded polymer fibre or jet is ejected towards the grounded collector. As the solvent-rich filament propels towards the grounded electrode, it undergoes significant whipping or bending instability that causes the diameter of the filament to decrease significant with a concomitant evaporation of the solvent^7,8^. The filaments that are deposited on the grounded plate exhibit a random orientation. Considering the application of electro-spun fibres for structural and functional applications, there is significant merit in producing fibres that are aligned. For example, in the context of using electro-spun fibres as reinforcements^9^, sensors^10^, actuators^11^ and electrodes^12^, controlling their spatial orientation during production is important^13,14^. Operations, during production or post-production, such as stretching the fibres to enhance the molecular orientation of the polymer chains requires the majority of the filaments to be oriented in one direction with minimal meandering or twisting.

A range of novel techniques have been demonstrated for producing aligned electro-spun fibres. A brief commentary is presented on each technique and their limitation when compared to the continuous haul-off method as described in the current paper. The fibre alignment methods reported in the literature can be classified under the following headings.

### Mechanical-based nano-fibre alignment methods

#### Rotating mandrel^15–17^

The use of a grounded rotating collector or mandrel, as opposed to a flat-pate collector as shown in Fig. 1a, is a popular method for producing aligned fibres. Due to the bending instability during electro-spinning, aligned fibres are obtained when the rotation speed of the mandrel is high enough to match the whipping speed of the polymer jet. The surface velocity reported for the mandrel range from between 3.5 and 12.3 m/s^15,16^. Although the rotating collector method is efficient at producing aligned fibres, extracting the fibres from the mandrel is not straightforward. Hence, operations such as secondary stretching and practical heat treatment at elevated temperature to enable carbonisation of the PAN fibres is not possible.

#### Rotating disk^18^

This is a variation on the rotating mandrel collector method and it involves the use of a rotating profiled edge disk^18^. The sharp edge provides a high localised electric filed strength and the fibres are attracted to this sharp rotating contour. The limitations of rotating mandrel mentioned above apply here and it is probable that some of the fibres will be deposited on the side wall of the spinning disk.

#### Rotating mandrel cage^19,20^

Instead of using a rotating mandrel, the collector is made up a series of bars that are arranged around the circumference of a pair of circular flanges with a specified distance between them. In effect, each pair of the grounded bars serve as a parallel electrode. It is claimed that aligned fibres could be achieved at lower rotation rates (~ 1 rpm) when compared to a conventional mandrel design that is operated at around 3000 rpm. A similar design was reported by Afifi et al.^20^. Whilst 60% of the fibres were found to be aligned within ± 5°, as in the previous case, the fibres cannot be extracted or subjected to any post-processing operations.

#### Rotating mandrel with insulating layers^21^

In this setup, two pieces of insulating tape is covered over the surface of the mandrel leaving a gap of 5 mm on the center of the mandrel; the fibres are deposited within the gap. 80% of the fibres were said to be aligned within 3° when the mandrel rotation rate was 3500 rpm. The limitations mentioned in the section above for rotating mandrels apply here with regard to continuous haul-off and post-processing. With reference to fibre alignment methods using mandrels, cages, etc., it is likely that the fibres will have an intrinsic curvature upon drying and this will not be conducive for the production of uniaxially aligned preforms.

#### Centrifugal alignment method^22,23^

Instead of using a rotating collector, here the spinneret is rotated and the fibres are deposited on to the surface of a disc or the inner surface of a cylinder to produce aligned fibres. The centrifugal force is responsible for the fibre alignment. It is assumed that with any of the mandrel-based collector approaches, the centrifugal fibre alignment method will also produce fibres with an intrinsic curvature. It is likely that it will be difficult to unspool the fibres as a continuous length.

#### Electro-spinning into a water bath with haul-off^24^

In this method, the electro spun fibres are collected in a liquid that effectively serves as a coagulation bath. The randomly-oriented fibres are extracted from the coagulation bath manually and wound on to a rotating mandrel. This method requires the solvent and the liquid that is used in the bath to be removed. This method is likely to enable continuous spooling after the initial manual operation. However, the generation of a yarn with a defined fibre orientation will be difficult. Moreover, secondary post-stretching will be required but due to the random orientation of the fibres in the yarn, it is likely to be difficult to control the relative dimensions along its length.

### Custom-designed electrodes

#### Auxiliary electrodes^25–28^

Auxiliary electrodes of specified shapes are positioned between the spinneret and the grounded electrode. Their basic function of the auxiliary electrodes is to modify the electric field between the spinneret and the grounded electrode. These authors used grounded rotating drums to achieve fibre alignment. They report that the presence of the auxiliary electrodes reduced the whipping of the polymer jet significantly. The degree of alignment reported was not specified. The limitations of the rotating method mentioned previously apply here.

#### Guard plate^29^

This method involved using conventional electro-spinning with a rotating mandrel but a brass guard plate (10 × 10 cm), with a small orifice in the middle, was threaded through the needle and positioned 30 or 20 mm behind the tip of the needle. 6% PEO/water–ethanol and 20% PS/DMF were electro-spun. They reported that the presence of the guard plate reduced the magnitude of the fibre whipping significantly. Moreover, the method was reported to be capable of producing 100% of highly-aligned fibres that were distributed within 4°. This was achieved when the surface rotation speed of the mandrel was around 3.5 m/s for PEO and between 4.0 and 6.1 m/s for PS/DMF. The problems associated with the rotating mandrel-based methods apply.

#### Parallel electrodes^30–32^

In addition to the use of rotating mandrel designs to produce aligned electro-spun fibres, the parallel electrode alignment method continues to be used extensively^33–35^. The parallel electrode method is relatively simple but yet extremely efficient in enabling fibre alignment. Instead of using a rectangular or circular grounded electrode, a pair of metallic grounded electrodes with an air gap between them is deployed. This was said to alter the electric field between the grounded electrodes which in turn enabled the polymer jet to swing back and forth between them^30^. The residual charge on the suspended fibres was reported to be responsible for repelling the incoming charged fibres. Whilst this method is elegant and efficient, it is only capable of producing short sections of aligned fibres. Furthermore, the fibres are aligned perpendicular to the grounded electrodes thus making spooling more complicated.

### Magnetic field-based alignment methods^36–38^

Instead of using positively charged auxiliary electrodes to influence the electric field between the spinneret and the grounded electrode, magnets are used. Magnets with the opposite poles facing each other were placed in between the spinneret and the grounded collector. It was suggested that the charged polymer jet experienced a radial Lorenz force as it travelled through the magnetic field^36^. The charged fibres were said to be attracted onto the collector and stretched across the gap of two opposite magnetic poles along the direction the direction that is normal to the surface of the magnets. However, it was pointed out that magnetic nanoparticles in the polymer solution are necessary in order to aligned the fibre with magnets^37^. In the context of manufacturing carbon fibres from PAN, the inclusion of magnetic nano-particles in not a viable proposition as it will compromise the desired mechanical properties. Furthermore, spooling the fibres is not convenient in this configuration.

### Insulating blocks^39^

Two insulating cubes are placed 5 mm apart between the needle and the grounded flat plate electrode. The working distance is normally 5 mm. The authors used modelling to show that the electric field strength between the insulating bocks was higher than that when they were not present. This was said to guide the polymer jet during electro-spinning whereby fibre alignment is enabled. The degree of fibre alignment was not reported. A key difference between this method and the Vee-shield technique is that the fibres in the latter case are aligned in the longitudinal direction of the grounded electrode and hence, enabling spooling or continuous haul-off.

### Near-field electro-spinning^40–43^

In conventional electro-spinning, the intrinsic bending instability resulting in the polymer jet whipping, after a short distance from the spinneret, is the major barrier for the production of aligned nano-fibres. Here, an elegant approach is used whereby the working distance is reduced to within the straight section of the polymer jet^42^. The added benefit of this approach is that it requires a lower applied voltage. Since the fibres are deposited on the grounded electrode before the commencement of whipping, the degree of molecular alignment that can be achieved is likely to be lower and the diameters of the fibres tend to be in the range 0.5–2 µm. The main advantage of this process is that the fibres can be positioned accurately on the grounded electrode. However, the process is not suitable for solvents with low volatility and the relaxation of the polymer chains in the solvent-rich jet is likely to be an issue if the intention is to manufacture reinforcing fibres with a high degree of molecular orientation.

### Oppositely charged needle electrodes^44^

These authors developed a process involving two spinnerets that were held horizontally and the needles were each charged at + 5 and − 5 kV respectively. The tips of the needles were separated by 14 cm. Details of the grounding arrangements were not specified. The electro-spun fibres from each spinneret were set to imping to form a cluster and this was presumably wrapped around a mandrel manually. They proposed that since the fibres emanating from each spinneret were charged oppositely, they adhere together. It is assumed that after the cluster of fibres are introduced to the mandrel and rotated, subsequent fibre production would result in oriented fibres as the mandrel is rotated. They proposed that the rotation speed of the mandrel had to be optimised for each polymer/solvent combination they investigated to prevent fibre fracture. The air turbulence caused by the rotating mandrel was reported to impede fibres orientation. The degree of fibre alignment was not specified.

### The need for spooling and fibre alignment

The ability to control the spatial orientation of the electro-spun fibres is desirable for a number of reasons. For example, with engineering materials such as fibre reinforced composites, the orientation of the fibres is a major factor that contributes to its overall strength and stiffness.

Aligning the fibres in one direction during electro-spinning can enable further axial stretching during spooling to enhance the degree of molecular orientation of the polymer chains. Applying a uniform tension to a bundle of electro-spun fibres during heat treatment will be necessary for precursors such as polyacrylonitrile (PAN) in order to compensate for shrinkage^45^. Applying uniform tension to the fibres will be simpler if they are oriented in one direction and plane. Moreover, aligned fibre stacks can be subjected axial stretching to control the residual fabrication stresses^46^. The mechanical testing of carbonised and graphitised fibres using conventional bundle-testing will be significantly simpler if the filaments are oriented in one direction^47–50^.

The ability to control the trajectory of the fibres as they are deposited will enable the production of unidirectional, cross-ply and quasi-isotropic fibre preforms. The technique can also be adapted for manufacturing processes such as filament winding, pultrusion and pre-pregging.

In the current paper, we report on a new technique for manufacturing highly-aligned PAN nano-fibres using DMSO as the solvent.

The current study is focused on PAN because it is the primary precursor that is used for the production of carbon fibres. The specific mechanical properties (property of interest divided by the density) of carbon fibres makes them extremely attractive for applications where the weight of the structure is important. Hence, advanced fibre reinforced composites continue to be used in industrial sectors such as aerospace, sports equipment, transport and civil engineering. A number of grades of carbon fibres are available commercially with specified Young’s modulus, tensile strength and failure strain. Whilst PAN-based carbon fibres have been researched extensively, areas where there may be scope to improve the tensile mechanical properties further for electro-spun fibres include: (1) developing techniques to increase the degree of molecular orientation^15,51–53^; and (2) reducing the diameter and flaw-density^52^.

On the first issue of increasing the molecular orientation, this can be achieved by:

- the intrinsic stretching during electro-spinning^54,55^
- using grounded collector designs such as a rotating mandrel^56^ and
- post-electro-spinning operations^57^.

However, since the solvent loading is generally in the range 70–80%, hence, the polymer chains can relax during the period they are on the grounded electrode, storage and subsequent heat treatment (for example to remove the solvent) resulting in a reduction in the degree of molecular orientation that was achieved during electro-spinning. Hence, the evaporation rate of the solvent, the crystallisation rate and dimension of the crystalline domains are important factors in addition to the uniaxial alignment of the electro-spun fibres^16,55,58^. In the context of the current article, the term “molecular orientation” is referred to the spatial arrangement of the polymer chains. The term “fibre alignment” is taken to represent the macroscopic orientation of the electro-spun fibres.

Optimising the above-mentioned parameters could lead to further improvements in the desired mechanical properties. Heat treatment of any solvent-based electro-spun fibres is of relevance as the solvent has to be driven out^59^; this even more important in the case of PAN prior to oxidation^58^.

The second issue mentioned above was the density of flaws in the fibres. It is generally appreciated that as the diameter of reinforcing fibres is reduced, the probability of encountering flaws will decrease in tandem^52^. Hence, with electro-spinning where fibres in the nano-metre diameter range can be produced, fibres with superior mechanical properties can be produced in theory^52^.

The selection criteria for the solvent is more straightforward in that it needs to comply with the following requirements: (1) dissolve the polymer; (2) evaporate during the transition from the Taylor cone trough the region of whipping and its deposition on the grounded collector; (3) enables the formation of a skin by the evaporation of the solvent from the surface of the fibre and/or by interacting with atmospheric moisture; and (4) ideally be non-toxic. The solvent selected in the current work was DMSO and it is fulfilled the above-mentioned requirements.

In the current study, the fibres were produced using the static and continuous haul-off methods in conjunction with the Vee-shield. The electro-spun fibres were evaluated using scanning electron microscopy and image analysis to estimate the degree of fibre alignment. The feasibility of manufacturing quasi-isotropic in situ nano-fibre preforms, without manual lamination, is demonstrated.

## Methods

### Dissolution of PAN

PAN powder with a weight average molecular weight of 230,000 g/mol was purchased from Goodfellow, UK. The solvent selected was 99.8% anhydrous dimethyl sulfoxide (DMSO) and it was purchased from Sigma Aldrich, UK and used without further purification. Due to the hygroscopic nature of DMSO, a silicone rubber septum was used to cap the container and the solution was withdrawn using a hypodermic needle and syringe. An advantage of DMSO over other solvents that can be used to dissolve PAN is that it is non-toxic. However, it has a boiling point of 189 °C. The PAN solutions were prepared in a 3-neck flask under reflux. A magnetic stirrer was used to agitate the polymer solution. The flask was flushed with dry nitrogen gas with a flow rate of 10 ml/h during the dissolution process. The refluxing was carried out for 6 h at 60 °C. The solution was filtered using 1 um pore size disposable syringe-filter (Xtra PTFE-100/25, CHROMAFIL, UK). The polymer concentrations made were 2, 4, 6, 8, 10, 12, and 14 wt/vol% in DMSO. The temperature of the solution was monitored using a sealed K-type thermocouple. After the refluxing period, the solution was permitted to cool to room temperature naturally whilst the flask was purges with nitrogen gas, after which, it was transferred to a glass vial and sealed until required.

### Characterising the polymer solutions

(1) Rheological properties: The viscosity of the PAN/DMSO solutions were determined on a cone-and-plate rheometer (Discovery Hybrid Rheometer, model HR-1, TA instrument, UK). The diameter of the plates was 40 mm with a cone angle of 4°. The rheometer was operated at 10 s^−1^ and the shear viscosity was obtained at 55 °C. (2) Electrical conductivity: The electrical conductivity of the polymer solutions was determined on a bench conductivity meter (model 4510, Jenway, UK). Before the measurements were undertaken, a sodium chloride conductivity standard solution (HI7033, Hanna Instrument) was used to calibrate the instrument. A temperature-controlled water bath was used to maintain the temperature during the measurements. The conductivity of each polymer/solvent system was measured at 55 °C and repeated three times to obtain an average.

### Electro-spinning apparatus

A schematic illustration of the conventional electro-spinning set-up is shown in Fig. 1a. The diameter and thickness of ground copper electrode were 10 cm and 1 mm respectively. The coded items in Fig. 1a–d are specified in the figure caption. The primary difference between the conventional electro-spinning setup and the Vee-shield methods, shown in Fig. 1a and b respectively, is the presence of a Vee-shaped fixture on the grounded electrode. Figure 1c shows a magnified view of the Vee-fixture.

With reference to Fig. 1a–c, the needle and grounded electrode were enclosed in a PMMA chamber of dimensions 400-width × 400-length × 400-height mm; the thickness of the PMMA sheet was 5 mm. The temperature inside the chamber was regulated using a 175 W infrared lamp (IR 175R E27 Infrared Bulb, Phillips, UK). The infrared lamp was situated on one side of chamber and it was left on during electro-spinning. A portable storage heater was used to pre-heat the chamber before the electro-spinning experiments; it was removed from the chamber just prior to electro-spinning. Silica gel was placed inside the chamber to lower the humidity to 20 ± 2%. The temperature within the chamber was maintained at 55 ± 2 °C. The temperature and humidity were monitored using a digital thermometer-hygrometer (RS Pro, RS Components, UK).

### Electro-spinning experiments

The PAN/DMSO solution was transferred to a 5 ml syringe (Terumo, UK) and it was secured to the liquid dispenser (NE-300, World Precision Instruments, UK). The liquid was dispensed at 0.01 ml/h. A 25-gauge needle (AD725025, Adhesive Dispensing Ltd, UK) with a bore and outer diameters of 0.3 and 0.5 mm respectively and length of 20 mm was attached to the syringe. A 1 mm metal ring of dimensions 0.5 mm ID, 0.8 mm OD and thickness of 1 mm was attached to the needle which in turn was connected to the positive output of the high-voltage DC power supply (73030, Genvolt, UK). The applied voltage was in the range 11–13 kV. The distance between the tip of the needle and the centre point of collector was set at 100 mm.

The electro-spinning experiments were initially conducted using the conventional electro-spinning setup (Fig. 1a) to optimise the processing parameters. Subsequent to this, the Vee-shield was placed and secured in position. In both cases, the electro-spinning was carried out for 30 min.

### Drying regime for the electro-spun fibres

The electro-spun fibres, with the cellulose substrate, were transferred to a tube furnace with a compressed-air flow of 10 cc/min from a gas cylinder. The sample was heated from ambient temperature at 2 K/min to 180 °C with a dwell of 6 h after which it was permitted to cool naturally to room temperature. The cellulose substrate was removed carefully and the fibres were stored in a desiccator until required.

### Characterisation of the electro-spun fibres

The morphology of the electro-spun fibres was characterised using a scanning electron microscope (HITACH TM-3030, Japan) where the instrument was operated at 15 kV. Prior to this, the samples were sputter-coated with gold/palladium alloy for 3 min using an Emscope SC 500 vacuum sputter-coater. Optical micrographs of the electro-spun fibres were captured using (Axioskop 2, Carl Zeiss, UK) with a digital camera (AxioCam HRc, Carl Zeiss, UK). Image analysis is used extensively to quantify the diameter^60–65^ of electro-spun fibres. Image J software (NIH, USA) was used to measure the diameter of the eletcro-spun fibres. During electro-spinning of each polymer solution, one batch represents five individual samples. Two samples were selected randomly and three micrographs were taken from three random spots from each of these sample. 100 measurements were taken from each micrograph; this represents 600 individual measurements per batch. The degree of fibre alignment for the PAN fibres obtained using the Vee-shield was also carried out using the ImageJ software (Version: 2.1.0/1.53c Build: 5f23140693 Copyright 2010–2021 (https://imagej.nih.gov/ij/)). The same protocol as that mentioned above for measuring the diameter of the fibres was used to quantify the relative orientation (600 measurements per batch of sample). The micrographs were imported into the ImageJ software to quantify the degree of alignment. The images were resliced to allow the border of the image to be parallel with the longitudinal edge of the substrate. A zero-degree reference line is drawn in the vertical plane that is parallel with the edge of the substrate. The orientation of the electro-spun fibre, in relation to the reference line, is obtained by drawing a line manually from one end of a fibre to the opposite end; this was performed for each of the randomly selected fibres and the data are logged within the ImageJ software.

A high-speed camera (Mini Ax 50, Photron, UK) was used to image the polymer jet during electro-spinning when using the conventional and Vee-shield setups. The high-speed camera was operated at 100,000 frames/second with a resolution of 300 × 300.

## Results and discussion

### Characterisation

The shear viscosity of the polymer solutions at 55 °C showed an exponential increase after the PAN concentration was above 6 wt/vol% in DMSO. The shear viscosity of the PAN/DMSO solution at 55 °C can be represented by a third order polynomial: y = 14981x^3^ − 1510.6x^2^ 45.474x − 0.1323. The exponential increase in the shear viscosity is attributed to the entanglement of the polymer chains above a certain concentration^66^. The surface tension and the electrical conductivity for neat DMSO at 55 °C are 37.92 mN/m and 6.5 µS/cm^2^ respectively. The surface tension and the electrical conductivity for the PAN/DMSO solutions at 55 °C was observed to increase as a function of the polymer concentration. The linear regression equations for the surface tension and the electrical conductivity of the PAN/DMSO solutions are y = 11.75x + 39.176 and y = 248.93x + 13.186 respectively; the corresponding coefficient of determination (R^2^) are 0.988 and 0.986 respectively.

### Fibre formation using the conventional electro-spinning setup

**Figure 2:**
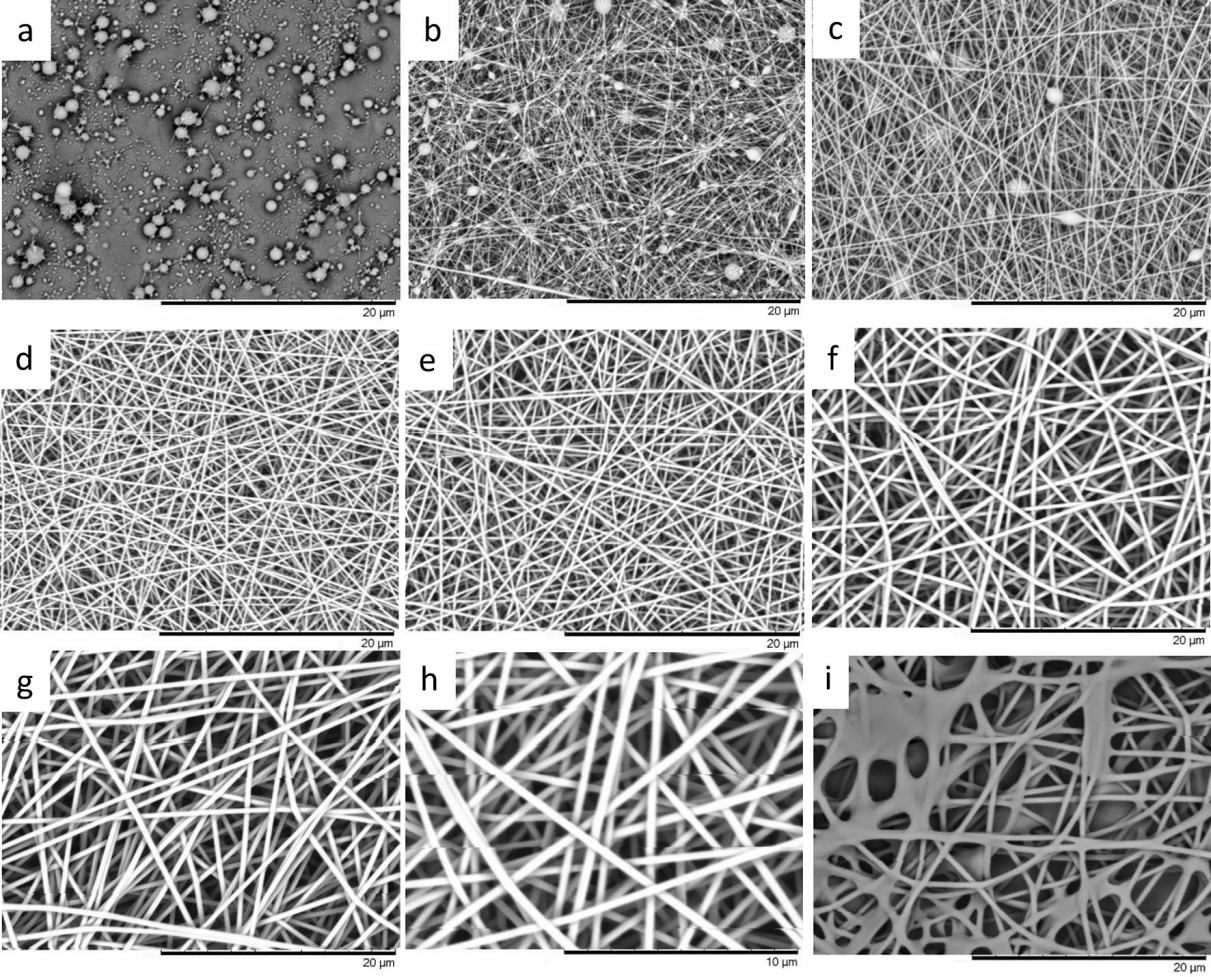
SEM micrographs of electro-spun fibers produced using solutions with polymer concentrations corresponding to (**a**) 2%; (**b**) 4%; (**c**) 6%; (**d**) 8%; (**e**) 10%; (**f**) 12%; (**g**) 14% wt/vol% PAN in DMSO that were electro-spun at 55 °C. (**h**) Represents a higher magnification of the 12% PAN solution that was electro-spun at 55 °C. (**i**) Corresponds to the 12% PAN solution that was electro-spun at 25 °C.

In the first set of experiments, the experimental setup shown in Fig. 1a for conventional electro-spinning was used to identify the optimum solution concentration and processing parameters. The assessment and acceptance criteria were that the electro-spun fibres had to be unfused, possess a circular circulation cross-section, be continuous and without beads. The optimum electro-spinning processing parameters were established experimentally to be a 12 wt/vol% PAN/DMSO along with an applied voltage of 13 kV and the solution feed-rate of 0.1 ml/h. The electro-spinning time was set to 5 min and the working distance was 100 mm. The temperature within the chamber was approximately 55 °C and humidity was 20%. The micro and macroscopic appearances of the electro-spun fibres, using the conventional experimental setup (Fig. 1a), as a function of the polymer concentration in DMSO are presented in Fig. 2a–g.

Figure 2a represents the case where the polymer concentration was 2 wt/vol% in DMSO. Evidence for electro-spraying is readily apparent where the diameters of the electro-sprayed particles ranged from 100 to 7000 nm (obtained over 600 measurements per sample). Figure 2b and c represent PAN concentration corresponding to 4% and 6% wt/vol in DMSO respectively. The beaded features observed in this micrograph are typical in appearance with conventional Plateau-Rayleigh instability. The spacing of the beads makes it unlikely to be due to the intermittent overflow of the pendant drop from the tip of the needle. In Fig. 2c as the concentration of the PAN is increased to 6 wt/vol% in DMSO, it is seen that the number of beads is reduced significantly when compared to Fig. 2b. The correlation between the viscosity of the polymer solution and the shear viscosity agrees with previous researchers that a critical viscosity is indeed required for fibre formation^67^. In addition, the ejected polymer jet needs to have the appropriate “strength” or viscoelastic properties to prevent the jet from fracturing as it undergoes whipping (bending instability)^68^. The majority of the fibres observed in Fig. 2c are in the diameter range 30–150 nm where beads were not observed. The average diameter of the fibres at the extremities of the elliptical drop are in the range 230–160 nm but they have a tapering profile away from the ellipse. The micrographs in Fig. 2d–h show fibres without beads and unfused fibres when inspected at two magnifications. The temperature within the chamber was identified as a critical parameter for electro-spinning PAN/DMSO solutions. This is because the boiling point of DMSO is 189 °C and its volatilisation is a prerequisite for the formation of a “skin” on the electro-spun fibre. The thickness of the outer skin on the fibres, as it is deposited on the grounded plate and the overall solvent content, will dictate if the circular cross-section of the fibre will be retained. In instances where the temperature is below a critical level (as a function of the processing conditions and environment), the fusion of the electro-spun fibres will be observed. This is illustrated in Fig. 2i where the fibres were electro-spun at 25 °C where significant fibre fusion is observed and this can be attributed to: (a) the excessive solvent that is retained in the fibre as it is deposited on the grounded electrode; and (b) the lack of a polymer skin on the fibres that assist with the retention of the circular cross-section. A summary of the measured fibre diameters shown in Fig. 2a-g is presented in Table 1; 600 measurements were taken per batch as described previously. From Table 1, an increase in the fibre diameter is seen with the polymer concentration. The frequency and density of probability plots for conventional electro-spinning (without the Vee-shield) is shown in Figure SM1 (Supplementary Material). The 2 wt/vol% PAN in DMSO has been excluded from the dataset as it represented electro-spraying (see Fig. 2a). The diameter of the electro-spun fibre is seen to increase as the concentration of polymer is increased. The fibre diameter distribution for the 12 wt/vol% PAN/DMSO is slightly broader when compared to the rest of the sample. Although the precise reason for this observation is not known at present, it may be attributed to small variations in the environmental conditions.

**Table 1:** Average fibre diameter for specified PAN/DMSO concentrations.

PAN concentration in DMSO (wt/vol%)	2	4	6	8	10	12	14
Average fibre diameter (nm)	54*	106*	142*	163	277	367	542
Standard deviation	0.13*	0.06*	0.03*	0.03	0.05	0.11	0.04

*Indicates beaded fibres.

### New fibre alignment method using the Vee-shield method: electro-spinning using the Vee-shield with a static substrate

**Figure 3:**
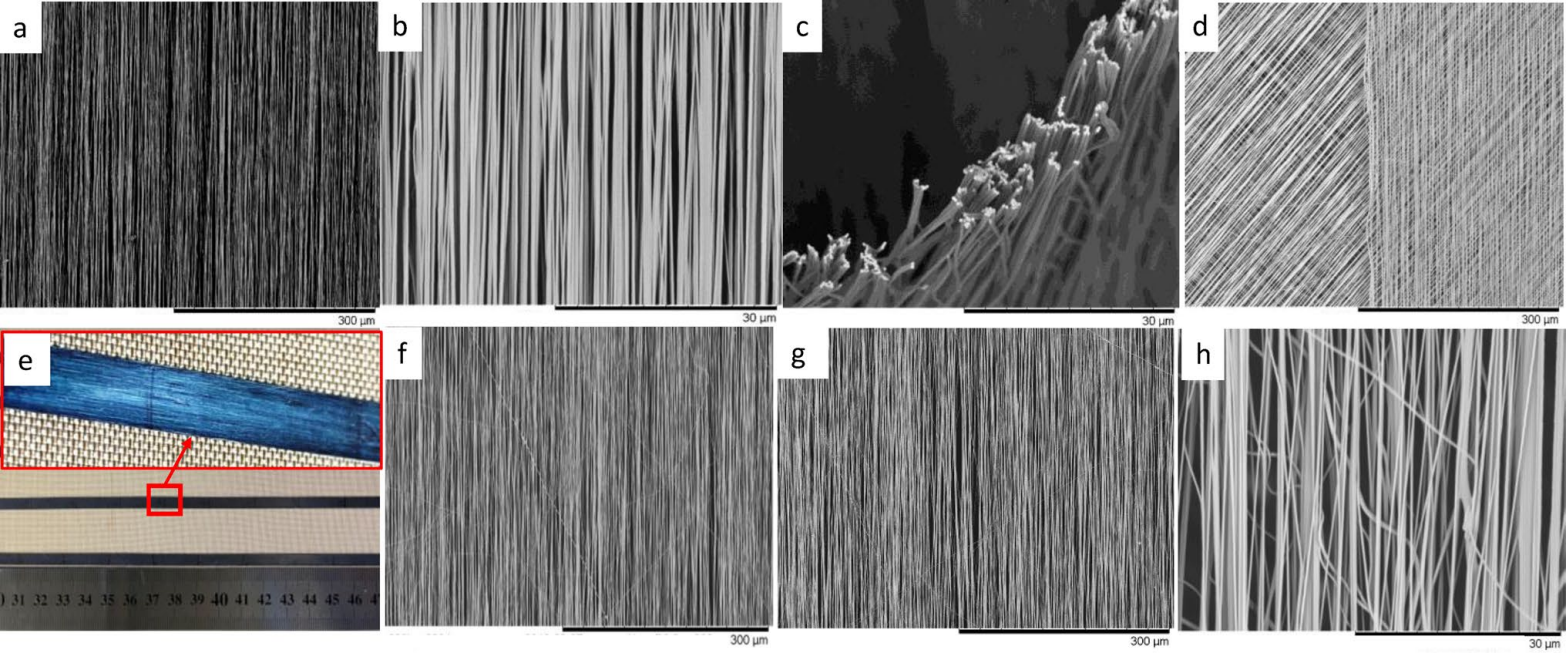
(**a**–**h**) Photographs and SEM micrographs showing electro-spun 12 wt/vol% PAN/DMSO that were produced when the cellulose substrate was static and spooled continuously using a motorised spooler and the Vee-shield fixture: (**a** and **b**) micrographs showing the degree of alignment achieved using the Vee-shield when the cellulose substrate was static; (**c**) transverse section of the electros-pun fibres after they were fractured in liquid nitrogen; (**d**) 0°/+ 45°/− 45° stacked arrays of aligned fibres; (**e**) a 5 mm wide strip of cellulose paper that was cut into short sections after spooling—the insert shows the electro-spun fibres that were deposited of the reel of cellulose paper; (**f**–**h**) represent SEM micrographs where the cellulose substrate was spooled continuously at 0.07 mm per minute.

With reference to the schematic illustration of the Vee-shield configuration shown in Fig. 1b and c, the opposite edges of the copper disk (items xvi and xvi’) act as the grounded electrodes. The role of the PTFE shield is discussed in the next section. With the aid of the high-speed camera, it was observed that the length of the straight section of the jet (before whipping was observed) was 1.7 cm for conventional electro-spinning and 3.6 cm when the Vee-shield was used. It will be shown later that the presence of the Vee-shield enables the polymer jet to oscillate between the ends of the grounded electrodes. Thus, the electro-spun fibres are deposited in an aligned manner on the narrower rectangular bottom section of the PTFE shield that is covered with a strip of cellulose paper (see Fig. 1c). The function of the cellulose substrate is to enable easy and controlled removal of the electro-spun nano-fibres. There is a gradual build-up of material at the edges of the grounded electrode and this can be reduced to some extent by hauling off the cellulose tape (discussed later). The degree of fibre alignment that was achieved with the Vee-shield design is shown in Fig. 3a and b at magnifications of 300× and 3000× respectively. Figure 3c shows a transverse section of the electro-spun fibres that were fracture in liquid nitrogen. The cross-section of the majority of the individual filaments was near circular with little evidence for any fibre fusion. The diameters of the fibres were in the range 250–600 nm with an average of 382 nm. The data on the fibre diameter were generated as described previously where a total of 600 individual measurements were made per batch and a summary is presented in Table 2 where over 90% of the fibres were aligned within three degrees.

**Table 2:** Distribution of fibre alignment achieved for 12 wt/vol% PAN in DMSO using the Vee-shield fixture with and without spooling.

Degree of fibre alignment	< 1°	1°–2°	2°–3°	3°–5°	5°–10°	10°–20°	20°–45°	45°–90°
Percentage of fibre distribution for the static setup (without spooling)	42.0	35.1	13.6	7.4	1.1	0.3	0.3	0.2
Percentage of aligned fibres with spooling	39.4	30.1	8.1	6.5	3.7	6.4	3.2	2.6

The standard deviations for the whole datasets for with and without spooling were 12.13 and 4.14 respectively.

The micrograph in Fig. 3d shows a stacked laminate of − 45°, + 45°, 0° aligned electro-spun fibres. Here, the Vee-shield was rotated manually to the appropriate angle to achieve the desired fibre orientation in relation to the longitudinal axis of the cellulose substrate. The degree of fibre alignment shown in Fig. 3d demonstrates that unidirectional, cross-ply quasi-isotropic laminates and preforms for the production of composites can be manufactured using the Vee-shield technique.

### Continuous electro-spinning and spooling using the Vee-shield method

The experimental setup shown in Fig. 1b can be changed easily from a static and batch production method to one where the aligned fibres are produced continuously, as shown in Fig. 1d where a motorised spooling rig was introduced. Here, a 12 wt/vol% of PAN/DMSO was electro-spun continuously onto a creel consisting of 100 cm of cellulose tape that was drawn at 0.07 cm/s and spooled. The nano-fibres produced using the Vee-shield and spooling (continuous hauling) are shown in Fig. 3e–h.

Figure 3e shows a 12 wt/vol% of PAN/DMSO that was electro-spun continuously and spooled; the insert shows a magnified view of a section of the cellulose reel with the aligned PAN fibres. Figure 3f–h represent SEM micrographs showing the degree of fibre alignment achieved during electro-spinning using the Vee-shield in conjunction with continuous spooling. Closer inspection of Fig. 3f and g show the presence of some fractured and misaligned fibres. This is reflected in the frequency distribution plots for the dataset from Table 2 are shown in Figure SM2 (Supplementary Material) where it is seen that the diameter distribution is narrower for the static Vee-shield when compared to continuous spooling. The full-width at half maximum (FWHM) from the histogram for the static and spooling methods are 11 and 31 respectively. Possible reasons for this observation include: (1) since the electro-spun fibres whip from one end of the grounded electrode to the other, as the cellulose substrate is hauled off, some of the fibres are unable to stretch as they are spooled and the they fracture; and (2) in order to prepare the electro-spun fibres for scanning electron microscopy, it was necessary cut the cellulose substrate into smaller sections. This resulted in some of the fibres becoming dislodged and misaligned. It was observed that the degree of fibre alignment during spooling was not equivalent to that obtained using the static electro-deposition method. The issue of the fibres fracturing during haul-off is shown at a higher magnification in Fig. 3h. It is envisaged that the number of fractured and those that cause misalignment as a consequence may be reduced by altering the manner in which the cellulose substrate is introduced to the slots in the copper electrode; this will be investigated and reported in due course.

It was observed that if the angle between the PTFE Vee-shield is altered from 60° degrees (optimal angle) to 0°, using the ground copper plate as the reference plane, under the same electro-spinning condition as mentioned previously, more than 70% the fibres were deposited on the surface of the PTFE shield as opposed to the on the cellulose tape. Moreover, the overall degree of axial fibre alignment was reduced significantly. However, when the angle was increased to 90° to the plane of the grounded copper electrode, fibres were not observed on the cellulose substrate, instead, they were deposited randomly in between the inner faces of the PTFE shield. The width of the rectangular section of the Vee-shield (see Fig. 1c) and the width of the cellulose substrate was also found to influence the degree of fibre alignment that could be achieved. For example, it was established that increasing the width from 5 to 20 mm led to a decrease in degree of fibre alignment along with the number of fibres deposited on the substrate.

As with any of the electro-spinning fibre alignment techniques, it is necessary to establish the fraction of the aligned fibres that are deposited where intended. In the Vee-shield method, when the angle was set at 60°, 53% of the fibres were deposited on the cellulose substrate after 30 min of electro-spinning. However, when the wall-angle of the PTFE shield was 0°, 30° or 90°, approximately 13, 26 and 1 wt% respectively were deposited on the cellulose tape. The data from this study are summarised in Table 3. The modelling discussed in the next section is used to explain this observation.

**Table 3:** Summary of the approximates weights of electro-spun nano-fibres that were deposited on the cellulose substrate and on the Vee-shield over 30 min.

PTFE shield angle (°)	Upper section (near the needle) (wt%)	Mid-section of the Vee-shield (wt%)	Lower section (near the substrate) (wt%)	On the cellulose substrate (wt%)
0	25 [0.71]	34 [1.30]	28 [0.84]	13 [1.14]
30	10 [1.30]	32 [0.89]	32 [1.67]	26 [1.51]
60	2 [0.55]	9 [1.14]	36 [1.10]	53 [0.55]
90	44 [1.14]	51 [2.77]	4 [1.79]	1 [0.55]

Another parameter that was found to have an influence on the degree of fibre alignment was the composition and properties of the Vee-shield material. It was seen that insulators such as PTFE, glass, polystyrene, polymethylmethacrylate have a positive impact of the degree of fibre alignment and density that could be achieved. This reiterates that the mode of operation of the Vee-shield where the whipping or oscillation of the electro-spun fibres is across the two edges of the grounded electrode.

The results of preliminary experiments to demonstrate the Vee-shield concept using different types of materials for the shield is presented in Table SM1 (Supplementary Material). The material used for the Vee-shield has to have a low relative permittivity and low volume resistivity. It is clear from this dataset that the ideal material for the Vee-shield is PTFE. Li et al.^61^ reached a similar conclusion with their parallel-plate design where the introduction of insulators in between the parallel-plates aided fibre alignment. The experimentally derived conclusion is that since electro-spinning is associated with charge transport, using conduction materials such as a metal is not an option for the Vee-shield material. Budi et al.^69^ used column guided arrays to produce aligned fibres. Accepting that it is not simple to extract these fibres for post-processing, they stated that using highly insulating materials or materials with a high dielectric constant for the air gap would increase the degree of fibre alignment.

In addition to electro-spinning PAN in DMSO, the following polymer were electro-spun using the Vee-shied method: (1) 30/70%PAN/lignin blend in DMSO; (2) 30% polyethylene oxide in deionised water; (3) 13.5% polyvinylpyrrolidone in ethanol; (4) 14% polyvinylidene fluoride in acetone/DMSO; and 20% polycaprolactone in acetone/DMSO. The purpose of this preliminary study was to demonstrate that the Vee-shield electro-spinning method is not limited to PAN/DMSO and that it could be used with other classes of polymers and solvents. The effectiveness of the Vee-shield technique for producing aligned fibres was demonstrated conclusively with the above-mentioned polymers and the data are presented in Table SM2. The properties of the polymers and the solvents used and the solution properties are presented in Table SM3. The outcome of these preliminary studies is discussed briefly.

With reference to Table SM2 (Supplementary Material), the electro-spinning parameters were similar for the polymers except for PAN where the temperature of the chamber was 55 °C. The electro-spinning time was 10 min in all cases and as before, 600 individual measurements were taken per type of polymer using ImageJ. The working distance was 10 cm and the applied voltage was 13 kV. The end-to-end distance between the grounded electrodes was 8 cm. The polymer solution dispensing rate was 0.1 ml/h. The humidity was maintained between 20 and 25% using silica gel within the chamber. The frequency plots for the diameter and degree of alignment for these combinations of polymers and solvents are presented in Figures SM3 and SM4 respectively. With reference to Figure SM3, the diameter of the electro-spun fibres and their respective standard deviations are shown in Table SM1 (Supplementary Material); the data for the 12 wt/vol% PAN in DMSO has been included to enable comparison. A notable feature is this dataset is that the range of diameters observed for PAN and PVDF is larger. It is not possible at this stage to assign a particular reason(s) for this observation as more detailed work is required. However, contributing factors could include the differences in the rate of charge dissipation, variations in the relative humidity, electrical conductivity of the solutions, the vapour pressure, rate of evaporation of the solvent etc. However, the data presented does demonstrate that the Vee-shield method can be used with most classes of polymers accepting that each will have to be optimised for the intended end-use application. A discussion on the differences between the Vee-shield method and template method is presented in the Supplementary Material section.

### Modelling

**Figure 4:**
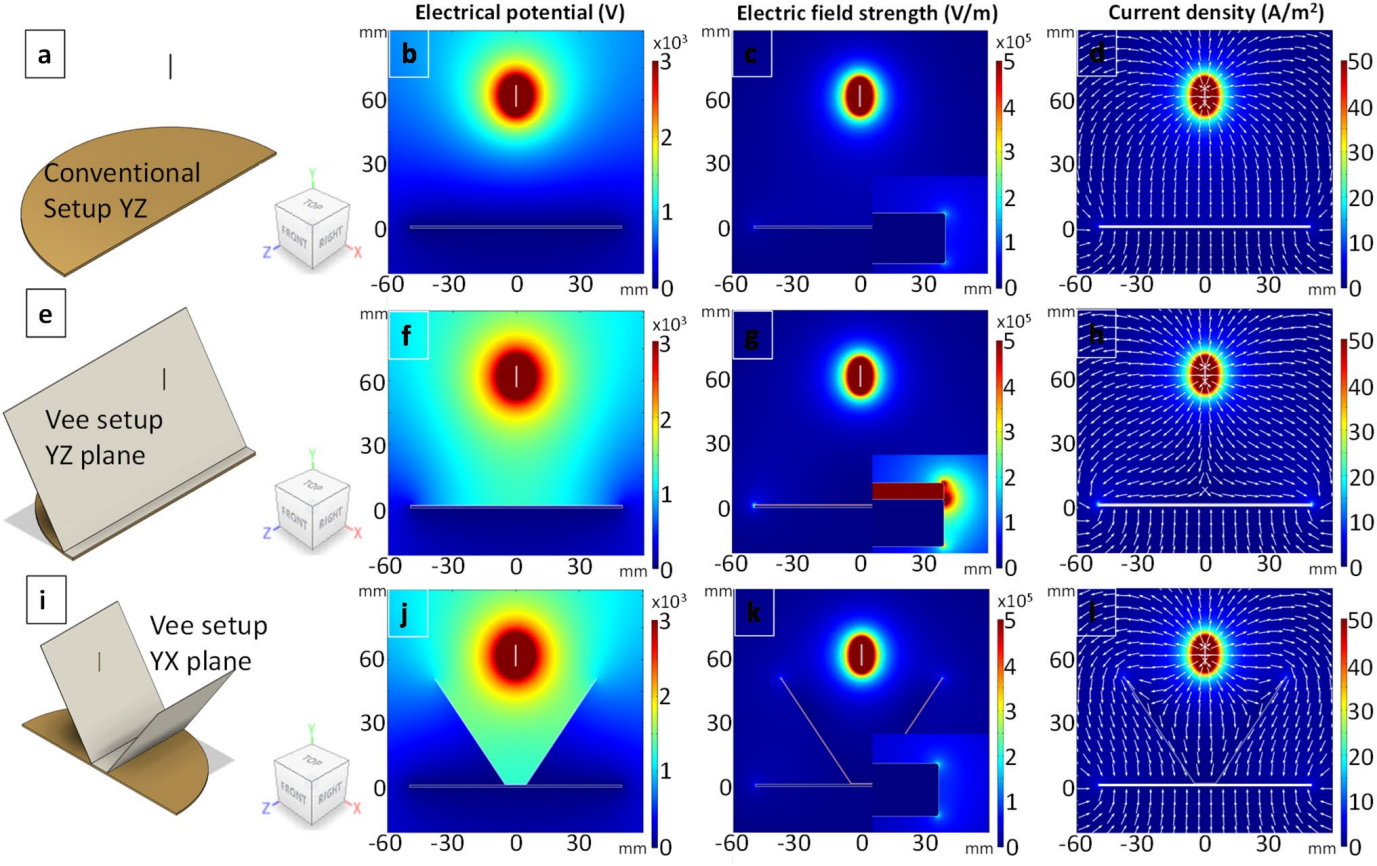
(**a**–**l**) The view planes for the conventional electro-spinning setup, the YZ and YX planes for the Vee-shield method are shown in (**a**), (**e**) and (**i**) respectively. The electrical potential, electric field strength and the current density are shown in the second, third and fourth columns respectively. The inserts in (**c**, **g** and **k**) show a magnified view of the extreme edge of collector and the electric field strength.

In order to investigate the mechanism responsible for fibre alignment using the Vee-shield method, COMSOL ACDC module with electric current physics was used^70,71^. Figure 4a, e and i show the relevant view planes and the coordinate system. The electrical potential, normalised electric field strength, normalised current density and current density vector plots are shown in the second, third and fourth columns respectively. Considering the electro-spinning setups shown in Fig. 4a—conventional and b, c—Vee-shield, the working distance and the applied potential for the simulations were set at 10 cm and 15 kV respectively. With reference to the simulation for the conventional setup (Fig. 4a), the electrical potential and the electric field strength are shown in Fig. 4b and c respectively. The current density plot shown in Fig. 4d indicates that that there is no preferential route for the electro-spun fibres to follow. Therefore, given the bending instability and whipping of the polymer jet, the fibres will be deposited randomly over a finite area, below the needle, on the grounded electrode.

The introduction of the insulating PTFE Vee-shield is shown in Fig. 4e–l is seen to have a significant influence on the electrical potential, normalised electric field strength, normalised current density and current density vector. It can be seen in Fig. 4f and j that the PTFE Vee-shield effectively confines the electric potential between the needle and the collector. Since the movement of charge is predominant along the direction with the higher potential difference, the charge carrier (polymer fibre jet in this case) will move along the YZ plan of the Vee setup. This is also seen in the electric field strength simulation (Fig. 4g, k) as well as the current density vector plots (Fig. 4h, l); the simulations support the observation that the electro-spun fibres oscillate predominantly between the exposed ends of the grounded electrode and thus aligned fibres are deposited along the length of the cellulose tape.

**Figure 5:**
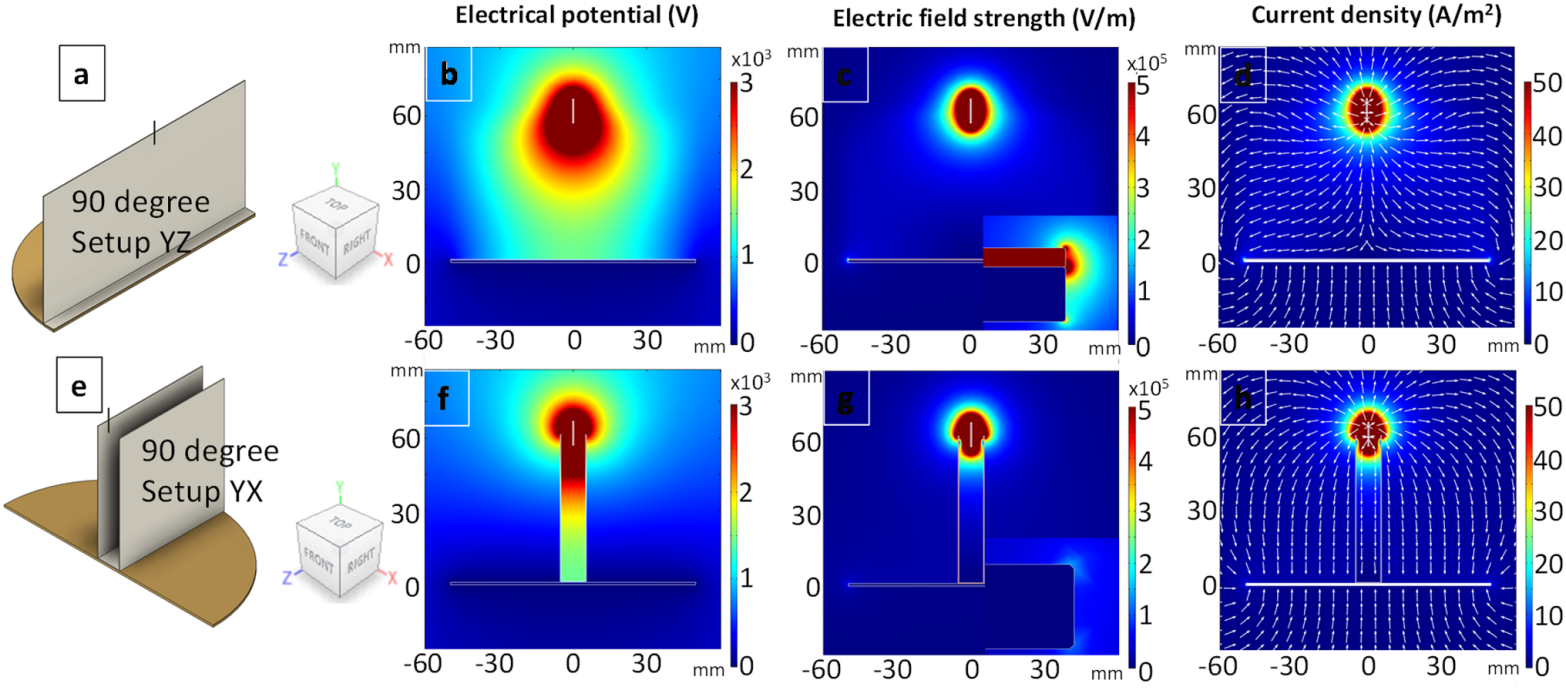
(**a**–**h**) Simulations showing the effect of changing the angle of the Vee-shield to 90°. The cross-sectional view planes are shown in (**a** and **e**). The simulations for the electrical potential, electric field strengths and current density in the YZ and YX planes are shown in (**b**–**d**) and (**f**–**h**) respectively. The inserts in (**c**) and (**g**) show the electric field strength at the extreme edge of the grounded collector where the red rectangle represents the PTFE.

Changing the angle of PTFE Vee-shield from 60° was observed to have a significant effect on the degree of alignment. For example, when the angle of the PTFE Vee-shield was 90° to the plane of the base as shown in Fig. 5a and e. Although the electric field strength is higher at the tip of the ends of the electrode in the YZ (Fig. 5c) than the YX plane (Fig. 5g), and the fact that the electrical potential is confined as seen in Fig. 5f, on inspecting the current density vector plots (Fig. 5d, h), it may be concluded that the fibres could be aligned along YZ plane. However, it is seen in Fig. 5b that the electric potential profile is wider near the tip of needle. This may explain the observation where the whipping of the fibre jet was seen to start closer to the upper section of the Vee-shield. This in turn led to an increase in the overall diameter of the whipping area and hence, the fibres (~ 99%) were deposited randomly in between the inner faces of the PTFE shield.

**Figure 6:**
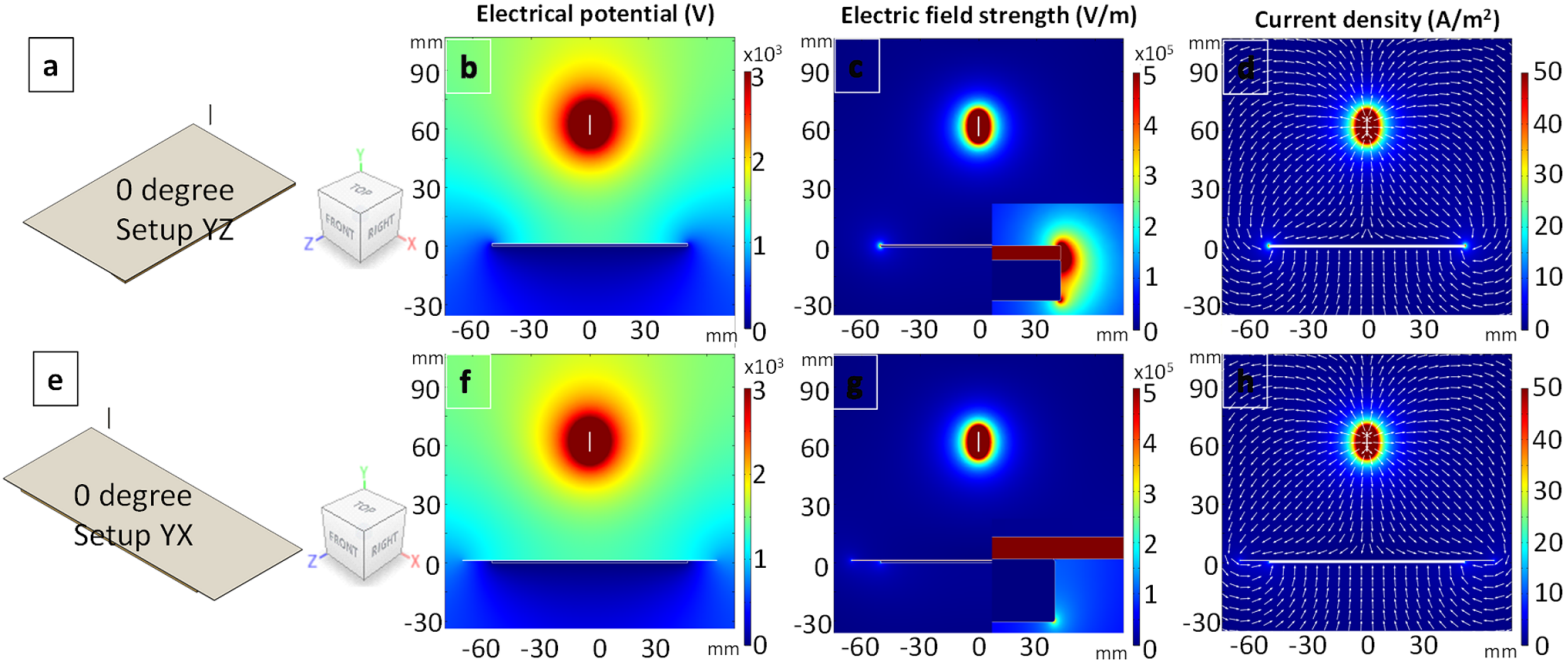
(**a**–**h**) Simulations showing the effect of changing the angle of the Vee-shield to 0°. The cross-sectional view planes are shown in (**a** and **e**). The simulations for the electrical potential, electric field strengths and current density in the YZ and YX planes are shown in (**b**–**d**) and (**f**–**h**) respectively.

When the angle is decreased to 0° as shown in Fig. 6, the current density vector plots (Fig. 6d, h) show that the charge can flow to the two both the ends. However, due to the presence of the PTFE plate and the fact that it is shorter along the YZ plane, the electric field strength is higher in this direction. On inspecting the electrical potential plots (Fig. 6b, f), the difference between the YZ and YX plane is not that obvious. Although the current vector plots suggest that the charge can flow to both the ends of the grounded plate, the density of the fibres that are deposited on the cellulose substrate will be significantly lower when compared to the case where the Vee-shield is at 60° as shown in Fig. 4; this is corroborated in the experimentally-derived data summarised in Table 3.

The data presented in Table 3 supports the simulations where it is observed that as the Vee-shield is changed from 90° to 0°, the mass of the electro-spun fibres that are deposited on the cellulose substrates increases progressively and reaches a maximum around 60° and then it decreases along with a decrease in the degree of fibre alignment. It is envisaged that further optimisation and modification of the Vee-shield, including the introduction of auxiliary electrodes will enable the majority of the electro-spun fibres to be deposited on the substrate.

A video of the continuous electro-spinning technique with the PTFE Vee-shield in conjunction with a PAN/DMSO solution is presented in the electronic Supplementary Materials section (Video_Continuous Electro-spinning and Spooling of PAN Nano Fibres).

## Conclusions

A new method, based on a PTFE Vee-shield, was developed and demonstrated to enable a 12 wt/vol% PAN solution in DMSO to be electro-spun to produce highly-aligned nano-fibres. The fibres were produced using a static and continuous haul-off method. In the static and spooling setups, 97% and 84% of the fibres aligned within 5° to an arbitrary vertical plane respectively. The introduction of the PTFE Vee-shield on the grounded conventional electro-spinning setup resulted in a modification of the electric field potential, electric field strength and the electric field vector; here, the intrinsic whipping of the polymer jet will oscillate between the ends of the grounded electrode thus producing highly-aligned nano-fibres. The Vee-shield was rotated manually to produce a stacked sequence of − 45°/+ 45°/0° without having to laminate the different layers with specified fibre orientations. There is significant scope to adapt this manufacturing technique for other classes of synthetic and bio-based polymers. Furthermore, the continuous nano-fibre production capability can be translated to manufacturing techniques such as filament winding, weaving, pre-pregging and pultrusion.

## Supplementary Information

Supplementary Information 1.

Supplementary Information 2.

## Data Availability

The material data reported here along with the associated protocols will be made available.
