# Supplementary material for: Electro-spinning of highly-aligned polyacrylonitrile nano-fibres with continuous spooling
Source: Sci Rep. 2021 Nov 5;11:21713. doi: 10.1038/s41598-021-99890-w (PMC8571412; doi:10.1038/s41598-021-99890-w)
Supplement: Supplementary file 1 — Supplementary Information 1. [file 41598_2021_99890_MOESM1_ESM.docx]

Supplementary Material

Figure SM 1(a-f) Electro-spun fibre diameter distribution for PAN at specified wt/vol% concentrations in DMSO using the conventional setup (without the Vee-shield). Typical SEM micrographs for this dataset are shown in Figure 2(b-g).

Figure SM 2(a & b) Histogram plots showing the fibre alignment distributions for the Vee-shield under static and continuous spooling conditions.

Figure SM 3(a-e) Electro-spun fibre diameter distribution for specified wt/vol% of polymer and solvent combinations that were produced using the static Vee-shield setup. The data for 12 wt/vol% PAN has been included in this dataset for comparison.

Figure SM 4(a-e) Degree of fibre alignment distribution for specified wt/vol% of polymer and solvent combinations that were produced using the static Vee-shield setup.

| Material used as the Vee-shield | Experimentally derived average % of aligned fibres between 0-5⁰  after 10 minutes | Experimentally derived aligned Fibre deposited on the substrate after 10 minutes of electro-spinning  (wt%) | Relative permittivity  @ 1MHz | Volume resistivity Ohm.cm |
| --- | --- | --- | --- | --- |
| No shield [1] | 0 | 0 | 1.0005 | 1 x 10^18^ |
| Wood  (Species unknown) [2] | 51  [5.65] | 22  [0.61] | 2 | ~3 x 10^17^ |
| Polystyrene [3] | 69  [2.79] | 42  [0.79] | 2.7 | 1 x 10^16^ |
| PTFE [3] | 78  [2.45] | 50  [0.51] | 2.1 | 1 x 10^19^ |
| Borosilicate glass [4] | 54  [4.98] | 32  [0.42] | 4.8 | 1 x 10^16^ |
| Aluminium [5] | 0 | 0 | - | 2.65 x 10^-8^ |

Table SM1 Summary of the experimentally derived data for the average of % of aligned fibres that were deposited on the substrate between 0-5 and the wt/vol% of the fibres that were were deposited on the substrate after electrospinning a 12wt/vol% solution of PAN in DMSO for 10 minutes. The relative permittivity and the volume resistivity for the Vee-shield materials are reported^1-5^.

|  | 30/70%PAN/  lignin | 30% PEO | 14% PVDF | 20%  PCL | 13.5% PVP | 12%  PAN |
| --- | --- | --- | --- | --- | --- | --- |
| Average fibre diameter (nm) | 352  [0.059] | 360  [0.064] | 1100  [0.206] | 311  [0.063] | 787  [0.076] | 367  [0.11] |
| % of fibre alignment  between 0-5⁰  after 10 minutes | 81  [2.03] | 82  [1.43] | 78  [1.68] | 70  [1.77] | 64  [2.09] | 78  [1.50] |

Table SM2 Experimentally derived data for the average fibre diameter and the % of fibre alignment achieved between 0-5⁰ after 10 minutes of electro-spinning. The values in parenthesis represent the standard deviation.

| Polymer | Mw | Solvent | Concentration (Polymer wt%) | Conductivity  (uS/cm) |
| --- | --- | --- | --- | --- |
| PVP | 1.3M | Ethanol | 13.5% | 12.5 ± 0.4 |
| PCL | 45k | Acetone/DMSO (2:1) | 30% | 0.55 ± 0.03 |
| PEO | 600k | DI water | 7.5% | 77.2 ± 0.8 |
| Lignin/PAN | 7,367^a^/230k (7:3) | Acetone/DMSO (2:1) | 24% | 168 ± 2 |
| PAN | 230k | DMSO | 12% | 18.9 ± 0.3 |
| PVDF | 534k | Acetone/DMSO (3:7) | 20% | 4.7 ± 0.08 |

Table SM3 Experimentally derived conductivity data for the polymer and solvent combinations investigated in this preliminary study. ^a^Inam Khan, PhD thesis, University of Birmingham, 2020.

References

1 Pawar, S., Murugavel, P. & Lal, D. Effect of relative humidity and sea level pressure on electrical conductivity of air over Indian Ocean. *Journal of Geophysical Research: Atmospheres* **114** (2009).

2 Brandrup, J., Immergut, E. H., Grulke, E. A., Abe, A. & Bloch, D. R. *Polymer handbook*. Vol. 89 (Wiley New York, 1999).

3 Serway, R. A. & Jewett, J. W. *Principles of physics*. Vol. 1 (Saunders College Pub. Fort Worth, TX, 1998).

4 Tropf, W. J., Thomas, M. E. & Harris, T. J. Properties of crystals and glasses. *Handbook of optics* **2**, 33.31-33.101 (1995).

5 Weatherwax, R. C. & Stamm, A. J. The electrical resistivity of resin-treated wood and laminated hydrolyzed-wood and paper-base plastics. *Electrical Engineering* **64**, 833-838 (1945).
